# diploS/HIC: An Updated Approach to Classifying Selective Sweeps

**DOI:** 10.1534/g3.118.200262

**Published:** 2018-04-06

**Authors:** Andrew D. Kern, Daniel R. Schrider

**Affiliations:** Department of Genetics, Rutgers University, Piscataway, NJ 08854

**Keywords:** Machine Learning, Deep learning, Selective Sweeps, Adaptation, and Population genetics

## Abstract

Identifying selective sweeps in populations that have complex demographic histories remains a difficult problem in population genetics. We previously introduced a supervised machine learning approach, S/HIC, for finding both hard and soft selective sweeps in genomes on the basis of patterns of genetic variation surrounding a window of the genome. While S/HIC was shown to be both powerful and precise, the utility of S/HIC was limited by the use of phased genomic data as input. In this report we describe a deep learning variant of our method, diploS/HIC, that uses unphased genotypes to accurately classify genomic windows. diploS/HIC is shown to be quite powerful even at moderate to small sample sizes.

An important goal of population genetics is to accurately identify loci in the genome which have undergone recent selective sweeps. In natural populations with complex demographic histories this is a difficult proposition, and thus much attention has been devoted to improving sweep-finding methods (*e.g.*, Nielsen *et al.* (2005); Lin *et al.* (2011); DeGiorgio *et al.* (2016)). The vast majority of this work has centered upon a “hard” sweep model of adaptation, wherein a *de-novo* beneficial mutation sweeps to high frequency carrying with it its linked genetic background (Smith and Haigh 1974; Kaplan *et al.* 1989). While such hard sweeps may be important sources of adaptive evolution, there is good reason to believe that selection from standing variation, a.k.a. “soft” sweeps, might be equally if not more important. Indeed, both theoretical and empirical evidence have accumulated sufficiently as to suggest that soft sweeps might be the more frequent mode of adaptation in many natural populations (Hermisson and Pennings (2005); Messer and Petrov (2013); Garud *et al.* (2015); Sheehan and Song (2016); Schrider and Kern (2017) but see Jensen (2014)). Thus if we wish to generate a more complete catalog of the genomic targets of selection, we need to be able to reliably identify both hard and soft selective sweeps.

While this sounds straightforward at first blush, a lingering issue is that hard sweeps can generate spurious signatures of soft and partial sweeps in regions that are at intermediate genetic distances from the true target of selection (Schrider *et al.* (2015)). This “soft-shoulder” effect thus requires any method developed for finding soft or partial sweeps to be aware of the genomic spatial context. We recently developed a method known as S/HIC that meets this criterion and is able to find both hard and soft sweeps in the genome with unparalleled accuracy and robustness (Schrider and Kern (2016)). S/HIC, like several contemporary tools for sweep finding (*e.g.*, Pavlidis *et al.* (2010); Lin *et al.* (2011); Ronen *et al.* (2013); Pybus *et al.* (2015); Sheehan and Song (2016)), utilizes supervised machine learning to classify individual windows of the genome as sweeps. In particular S/HIC summarizes a genomic window on the basis of a large vector of transformed summary statistics and then uses those “features” as input to an Extra-Trees classifier (Geurts *et al.* (2006)). The feature vector of S/HIC captures spatial patterns of variation across a region of the genome by recording the relative values of a given statistic in subwindows as a vector. While S/HIC performs well both on simulated and empirical data, its use is currently limited to population genomic samples with phased genotype data as it relies on haplotype-based statistics.

Here we introduce a new version of S/HIC that alleviates this issue that we call diploS/HIC (pronounced “deep-lo-shick”). We outline a series of summary statistics which we calculate on unphased genotypes across a genomic window which allow for accurate classification of genomic windows in the context of supervised machine learning (c.f. Schrider and Kern (2018)). diploS/HIC uses a deep convolutional neural network (CNN) approach to classification (LeCun *et al.* (2004); Krizhevsky and Hinton (2010); Krizhevsky *et al.* (2012)) whereby we represent as an image the multidimensional vector of statistics calculated from the window to be classified. Using simulation we show that diploS/HIC retains nearly all of the power and accuracy of S/HIC. Moreover we show that convolutional neural networks outperform our original Extra-Trees classifier both for our original S/HIC formulation and diploS/HIC.

## Methods and Model

### Coalescent simulation

diploS/HIC is a supervised machine learning classifier that uses coalescent simulation to generate training data. All of our simulations in this paper are performed using discoal (Kern and Schrider (2016)), though other simulators could be used provided they are flexible enough to simulate the evolutionary scenarios described below. We simulated under a number of demographic scenarios and selective scenarios both with constant population size and with the very strong population growth we observed in *Anopheles gambiae* from Burkina Faso (Anopheles gambiae 1000 Genomes Consortium (2017)). These simulations were performed under five sample sizes, n={20,40,60,80,100} haploid chromosomes. Command lines for each scenario are given in Table S1. For simulations with selective sweeps we sampled from a uniform prior of population scaled selection coefficients (α=2Ns). For each combination of sample size, demographic history, and rage of *α*, we simulated large chromosomal regions that we later split into 11 subwindows (as in Schrider and Kern (2016)). We simulate hard sweep training examples by generating $2\times 10^{3}$ simulations where we conditioned upon a fixation in the exact center of our region (*i.e.*, the middle of the 6^th^ subwindow). Linked-hard training examples are generated by simulating $2\times 10^{3}$ sweeps where the selected site is at the center of each of the remaining 10 subwindows flanking the central window. For soft sweep and linked-soft training examples we follow this same procedure but add a uniform prior on the frequency, *f*, at which a mutation is segregating at the time it becomes beneficial such that f∼U(0.0,0.2). Most simulations under constant population sized varied the time of fixation of the beneficial allele, *τ*, over a uniform range τ∼U(0,0.025) in units of 4N generations, although where noted we fix τ=0. Our simulations using *Anopheles gambiae* demography were done with a more recent distribution of fixations times, τ∼U(0,0.0004). Finally we generate $2\times 10^{3}$ simulations without sweeps but with the same demographic history as selected training examples.

From these simulations a balanced set with equal representation of all five classes (neutral loci, hard sweeps, soft sweeps, loci linked to hard sweeps, and loci linked to soft sweeps) to be used for machine learning is created through sampling without replacement. This constitutes the training set for our classifier. The simulation procedure is then repeated to generate an independent test set of $≳10^{3}$ examples per class on which to benchmark trained classifiers.

### The diploS/HIC feature vector and associated summary statistics

Our original version of S/HIC used as a feature vector the transformed values of 11 summary statistics calculated in 11 sub-windows centered upon the window to be classified (Schrider and Kern (2016)). These statistics were *π* (Tajima (1983)), ${\hat{\theta }}_{w}$ (Watterson (1975)), Tajima’s *D* (Tajima (1989)), ${\hat{\theta }}_{H}$ and Fay and Wu’s *H* (Fay and Wu (2000)), the number of distinct haplotypes, average haplotype homozygosity, $H_{12}$ and $H_{2}/H_{1}$ (Garud *et al.* (2015)), $Z_{ns}$ (Kelly (1997)), and the maximum value of ω (Kim and Nielsen (2004)). These statistics are then normalized across subwindows to capture the relative shape of a given statistic across the larger region such that the value of some statistic *x* in the *i*th subwindow is $x_{i}=x_{i}/\sum_{j}x_{j}.$ If the minimum value of *x* is negative, the elements of the vector are rescaled such that $x_{i}=x_{i}-min\left(x\right).$ Classification of genomic windows then proceeds using an Extra-Trees classifier (ETC), and windows were identified as belonging to one of five classes: a hard sweep, a soft sweep, a window linked to a hard sweep, a window linked to a soft sweep, or a “neutral” window unlinked to a sweep. Given our choice of summaries, S/HIC relies upon phased haplotype data (*e.g.*, $H_{12},$ $Z_{ns}$) and a polarized site frequency spectrum (SFS) (Fay and Wu’s *H* and ${\hat{\theta }}_{H}$), though the latter can be removed with little loss of power (data not shown).

To relax these constraints we set out to design a feature vector that would allow for genomic windows to be classified into our five classes on the basis of a set of statistics that could be calculated from unphased genotype data. In particular we aim to capture quantities that describe three axes of the data: the SFS, haplotype structure in the region (we will only have partial information about this), and linkage disequilibrium (LD).

Unphased data do not lead to difficulties in summarizing the SFS, so we use three summary statistics: *π* (Tajima (1983)), ${\hat{\theta }}_{w}$ (Watterson (1975)), and Tajima’s *D* (Tajima (1989)). Describing the haplotype structure and LD from unphased data are more difficult. Let the genotype vector of the *k*th individual be $x_{k}=\left\{x_{k0},x_{k1},\ldots ,x_{km}\right\}$ where individual *k* has been genotyped at *m* loci and $x_{ki}\in \left\{0,1,2\right\}$ according to the number of non-reference (minor) alleles individual *k* has at locus *i*. We define the genotype distance, $g_{kl},$ between two individuals *k* and *l* such that $g_{kl}=\sum_{i=0}^{m}1_{x_{ki}\neq x_{li}}.$ We note that $g_{kl}$ is an underestimate of the true sequence divergence, and thus only provides a lower bound on haplotype distance. While that is so, moments of the distribution of pairwise $g_{kl}$ comparisons for a sample prove informative for differentiating among our sweep and neutral classes of loci. In particular we use the variance, the skewness, and the kurtosis of the $g_{kl}$ distribution as summary statistics.

**Figure 1:**
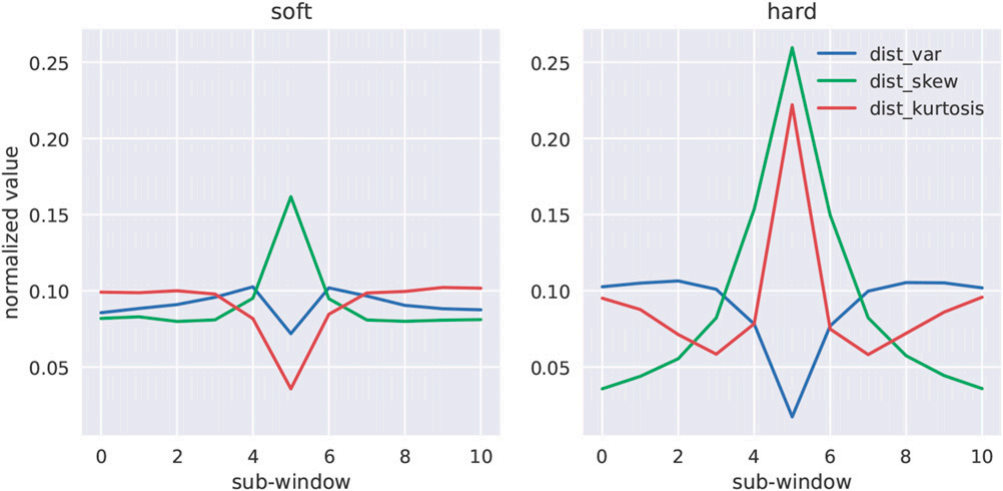
Genotype distance distribution summaries for soft and hard sweeps. We simulated $2\times 10^{3}$ sweeps under constant population size with moderately strong selection (α∼U(250,2500)), in which the fixation of the beneficial allele happened immediately prior to sampling (*i.e.*, τ=0). The relative magnitude of the variance, skew, and kurtosis of the distribution of pairwise genotype distances, $g_{kl},$ across sub-windows of a larger simulated region is shown for soft and hard sweeps respectively.

Figure 1 shows mean values of the normalized subwindow $g_{kl}$ distribution moments from constant size population simulations of hard and soft sweeps (see figure legend for details). While each of these moments contains information about the location of a sweep, the kurtosis in particular adds information as to whether a given sweep is soft *vs.* hard. Here we are using this for diploid genotype data, however we note that a similar summary of haploid data (*i.e.*, the higher order moments of haplotype distances) might add excellent information for sweep classification from phased data.

**Figure 2:**
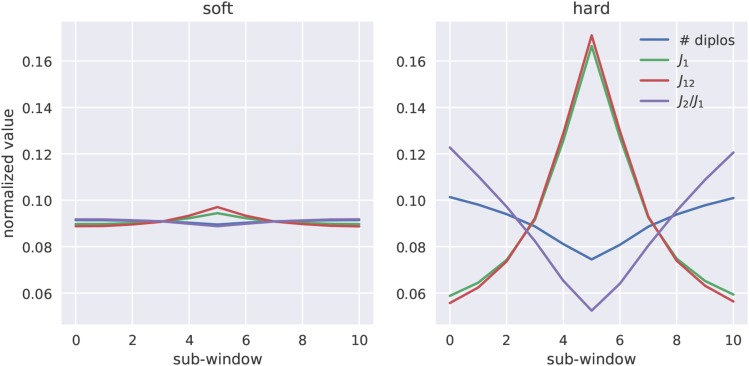
Multilocus genotype frequency spectrum statistics for soft and hard sweeps. We simulated $2\times 10^{3}$ sweeps under constant population size with moderately strong selection (α∼U(250,2500)) and in which the fixation of the beneficial allele happened immediately prior to sampling (*i.e.*, τ=0). The relative magnitude of the number of diploid genotypes, $J_{1},$ $J_{12},$ and $J_{2}/J_{1}$ across sub-windows of a larger simulated region is shown for soft and hard sweeps, respectively.

We also summarized the frequency distribution of multilocus genotypes in a manner analogous to the haplotype frequency summary statistics of Messer and Petrov (2013) and Garud *et al.* (2015). The simple idea here is to treat the multilocus genotype as a population genetic entity, sometimes referred to as the “diplotype”. We recorded four summaries: the number of distinct genotype vectors, and then the multilocus genotype equivalents of $H_{1},$ $H_{12},$ and $H_{2}/H_{1},$ call them $J_{1},$ $J_{12},$ and $J_{2}/J_{1}.$ The normalized values of $J_{1},$ $J_{12},$ and $J_{2}/J_{1}$ across simulated genomic regions that contain either a soft or hard selective sweep are shown in Figure 2. Each of these statistics adds useful information for detecting hard sweeps, however they are less sensitive to soft sweeps than the $g_{kl}$ distribution introduced above. Thus, these statistics may be useful for discriminating between hard and soft sweeps. Finally, to summarize patterns of linkage disequilibrium we computed the equivalents of $Z_{ns}$ (Kelly (1997)) and *ω* (Kim and Nielsen (2004)) using genotypic LD rather than gametic LD calculated according to Rogers and Huff (2009).

Thus our complete feature vector for diploS/HIC consists of 12 summary statistics calculated in each of our 11 sub-windows (132 in total): *π*, ${\hat{\theta }}_{w},$ and Tajima’s *D*, the variance, the skew, and the kurtosis of $g_{kl},$ the number of multilocus genotypes, $J_{1},$ $J_{12},$ $J_{2}/J_{1},$ unphased $Z_{ns},$ and the maximum value of unphased *ω*.

### Sweep finding as image recognition

**Figure 3:**
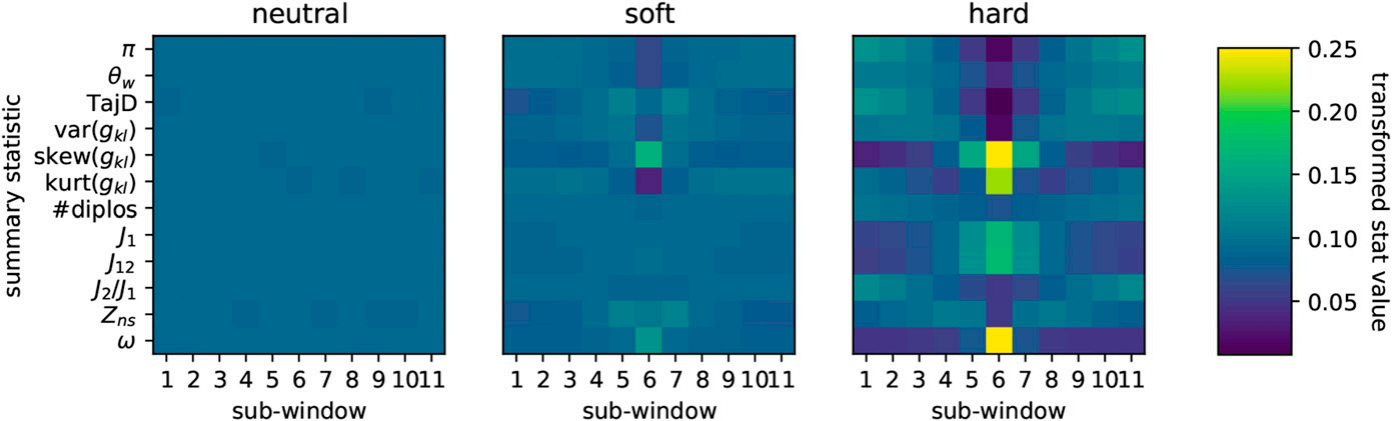
Average-case images for neutral regions, soft sweeps, and hard sweeps. Here we show the average images for three classes of training data, neutral regions, soft sweeps, and hard sweeps, that are used as input to our convolutional neural network for classification. Individual subwindows of the region to be classified are shown in columns. Rows represent each of the 12 summary statistics (see text). The upper end of the color scale has been truncated at 0.25 for visualization. These two images represent the average image from a set of $2\times 10^{3}$ simulations with parameter values as described in Figure 1.

In our original implementation of S/HIC we used an Extra-Trees classifier to classify genomic windows on the basis of a large vector of summary statistics that had been transformed to capture spatial information. While the classifier was thus able to use spatial information, the actual implementation was completely naive to any notion of spatial structuring among subwindows. In an effort to utilize spatial relationships of summary statistics among windows more effectively, we reasoned that we might be able to represent our transformed summary statistics as an image that we could then train a convolutional neural network (CNN) to recognize. As before, we record our vector of 12 summary statistics across 11 subwindows in which our central window is the one to be classified. Putting summary statistics on rows and subwindows on columns we then arrive at an image representation of a locus on the basis of our collection of summary statistics (Figure 3). In Figure 3 we present averages across each class and in Supplemental Figure S1 we show examples of several individual simulation images; note that any given individual realization shown in Supplemental Figure S1 can differ substantially from the mean. These are the raw material that are used for training our CNN classifier.

### Convolutional Neural Network

To classify genomic regions into one of our five classes (a hard sweep, a soft sweep, a window linked to a hard sweep, a window linked to a soft sweep, or a “neutral” window unlinked to a sweep) diploS/HIC uses a deep convolutional neural network (CNN) trained from simulation data. CNNs are characterized by having one or more “convolutional” layers in their networks whose purpose is to capture essential features of an image by sliding a receptive field of specified size across the image an then computing dot products between the original image values and that of the convolutional filter. CNNs are incredibly powerful tools for image recognition because they reduce the number of parameters necessary for deep learning on images while maintaining enough of the complexity of the original data for accurate classification or regression (LeCun *et al.* (2004); Krizhevsky and Hinton (2010); Krizhevsky *et al.* (2012)).

**Figure 4:**
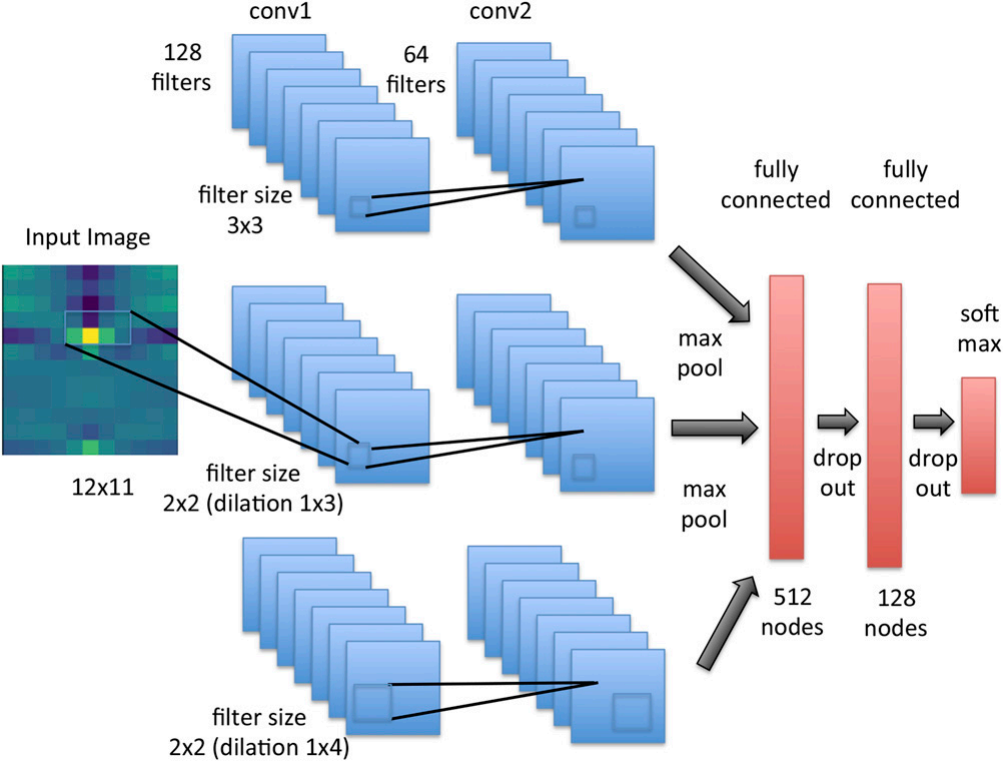
Convolutional neural network structure of diploS/HIC. The CNN takes a single image as input and then passes that image to three three convolution layer units each with different filter sizes to capture variation in the image at different physical scales. Each convolution unit consists of two convolution layers followed by a max pooling and a dropout layer. The outputs from the convolutional units are then concatenated and fed to two fully connected dense layers, each themselves followed by dropout. Finally a softmax activation layer is applied to get a categorical classification.

diploS/HIC uses a CNN architecture that attempts to capture relationships between windows at multiple physical scales. To do this our input image is used as input to three different branches of a CNN, each of which has two dimensional convolution layers with different sized filters (see Figure 4). Each of these convolutional layers slides a “receptive filter,” basically a focal window, across the input image, taking dot-products between filter weights (*i.e.*, the fitted parameters of the network layer) and the input itself. Doing this at multiple scales simultaneously allows us to capture how summary stats are responding across the physical chromosome more effectively. The top-most “convolutional unit” has two 3×3 convolutional layers, where as the other two convolutional units have 2×2 filters that are dilated such that the receptive filter is rectangular–the middle unit uses a 1×3 dilation and the bottom unit uses a 1×4 dilation (*c.f*. Yu and Koltun (2015)). In experiments that we do not show here, this structure was found to outperform simpler CNNs that did not attempt to capture multiscale information. Moreover, while the two dimensional convolution structure implies that the summary statistic row ordering might matter, we experimented with different permutations and observed no discernible effect on accuracy. Indeed removing any dependence of row order by changing the convolutional filters to sizes of 12×3 and 12×2 respectively yields no difference in performance (Table S2). Each of the convolutional units used consists of two convolutional layers with ReLu activations, followed by a max pooling layer (to reduce the dimensionality of the representation), which itself is followed by a dropout layer to control overfitting by regularizing the network through setting a subset of weights to zero stochastically. This combination of convolutional layers followed by pooling and dropout layers is quite standard in image recognition (see O’Shea and Nash (2015) for an approachable introduction). At this point in the network the outputs from all three convolutional units are flattened and concatenated and then fed to a series of two fully connected dense layers, between which we again utilize dropout layers, before finally arriving at a softmax activation layer that outputs categorical class membership probabilities (Figure 4).

We trained the diploS/HIC CNN using simulations generated as described above. In general both training and independent test sets consisted of $2\times 10^{3}$ simulations per class unless otherwise specified. Optimization of the CNN parameters was performed using the Adam algorithm (Kingma and Ba (2014)) tracking as our objective function the accuracy of the classifier on a validation set. Optimization epochs were run until the validation accuracy stopped improving beyond a difference of 0.001 between epochs. In practice this training was quite rapid, with generally less than 20 epochs needed until stopping.

We implement our CNN using the open source package TensorFlow (Abadi *et al.* (2016)) and its associated higher level python API Keras (Chollet *et al.* (2015)).

### Data Availability

Our software for performing the diploS/HIC feature vector calculation and deep learning is available from https://github.com/kern-lab/. Supplemental material available at Figshare: https://doi.org/10.25387/g3.6097337.

## Results

**Figure 5:**
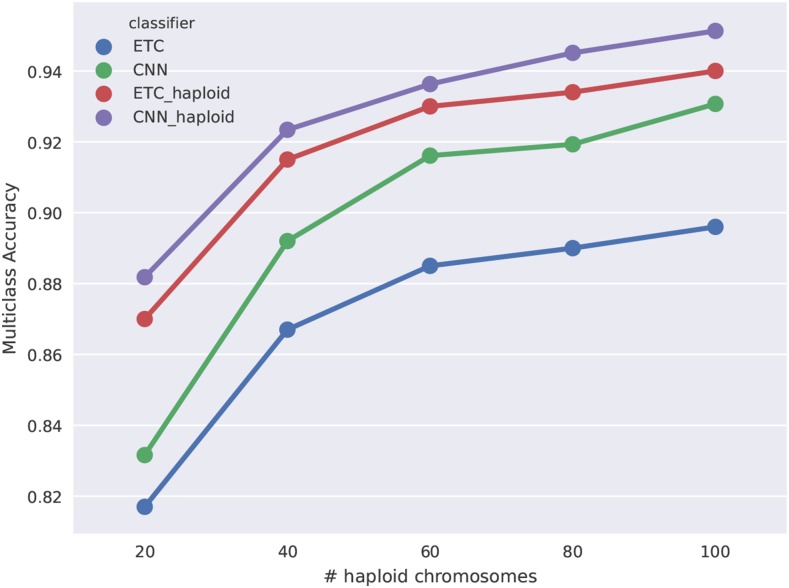
Multiclass classification accuracy for diploS/HIC and related classifiers. Here we compare the multiclass classification accuracy of four related classifiers across a variety of sample sizes: the original S/HIC classifier (“ETC_haploid”), a S/HIC classifier which uses a CNN rather than an ETC (“CNN_haploid”), our new classifier for unphased data trained with an ETC (“ETC”), and finally our new classifier training with a CNN (“CNN”). Note the y-axis has been truncated to better display the separation between methods.

We were first interested in examining how using a CNN with diploS/HIC affected classification accuracy in comparison to the Extra-Trees classifier we had used with S/HIC. For this we generated constant population size simulations with moderately strong selection, α∼U(250,2500), where the beneficial mutation had fixed immediately prior to sampling (*i.e.*, τ=0) across five different sample sizes of n={20,40,60,80,100} haploid chromosomes. The complete parameterization of these simulations is given in Table S1. We were also interested in characterizing the difference between using unphased and phased data in both S/HIC implementations. Our expectation was that phased data should add significant information to the classification task we are interested in. Figure 5 shows accuracy of four classifiers as a function of sample size. Those classifiers are the original S/HIC classifier (labeled “ETC_haploid”), a CNN classifier which uses the original S/HIC feature vector (“CNN_haploid”), and our new, unphased data feature vectors trained with either a CNN (labeled “CNN”) or an ETC (“ETC”). A few trends are notable. First, it is clear that phased data add useful information for classification– both of our phased (haploid) classifiers are more accurate than our our unphased classifiers. Second, our unphased classifiers themselves are performing quite well even at small sample size (for instance ∼83% accuracy at n=10 diploid individuals). Third, the convolutional neural network approach adds a bit of accuracy, although the gains aren’t dramatic. While that is so, the size of the training set used here, 2000 examples per class, is quite small for deep learning, thus we might expect to see a greater difference between the CNN and the ETC approaches if we were to increase the size of our training set. These trends generalize to stronger and weaker selection although accuracy varies across these cases (see Figures S2 and S3).

**Figure 6:**
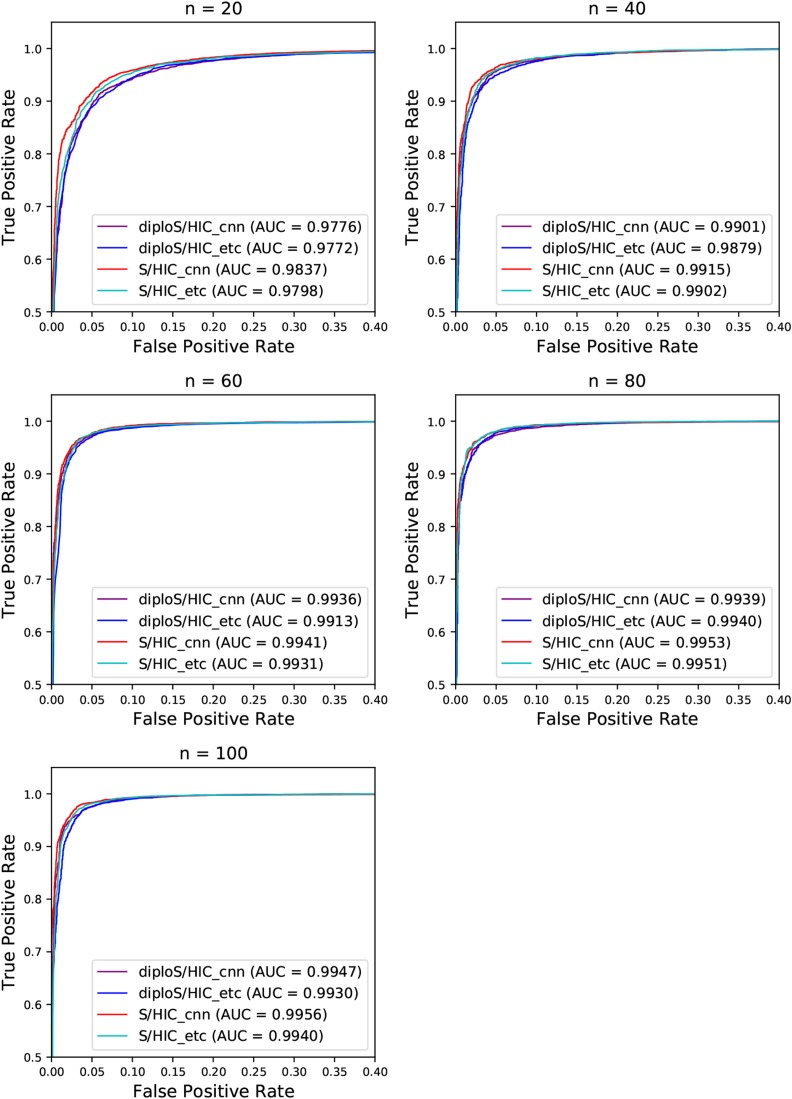
ROC curves showing the true and false positive rates for various classifiers. Here we show ROC curves for the binary classification task of Hard and Soft *vs.* linked and neutral regions. ROC curves are shown for both unphased and phased data classifiers as well as for the two machine learning algorithms considered. Each panel represents a different sample size of haploid chromosomes from n={20,40,60,80,100}. Note the scale of the x and y axes does not run from (0,1) but instead is zoomed in to show what little separation among methods there is.

We next turn our attention to a slightly more realistic scenario in which the beneficial mutation of interest has not fixed immediately before sampling the population, but instead fixed within some uniform range of time before sampling. We generated simulations as before, under constant population size and moderately strong selection, α∼U(250,2500), but allowing the time of fixation, τ, to vary such that τ∼U(0,0.025). To characterize performance of the classifier under this regime, in Figure 6 we show ROC curves (*i.e.*, true positive *vs.* false positive rates) for our classifiers’ abilities to distinguish between sweeps, both hard and soft, *vs.* unselected regions, in this case both neutral and linked regions. At all sample sizes we have excellent discriminatory power for this binary classification task. This is also the case for stronger and weaker selection (Figures S4 and S5). Across each of these scenarios considered diploS/HIC is surprisingly competitive with our original, phased data implementation. This suggests that for sweep finding diploS/HIC will be a very useful tool in datasets without information on phase.

**Figure 7:**
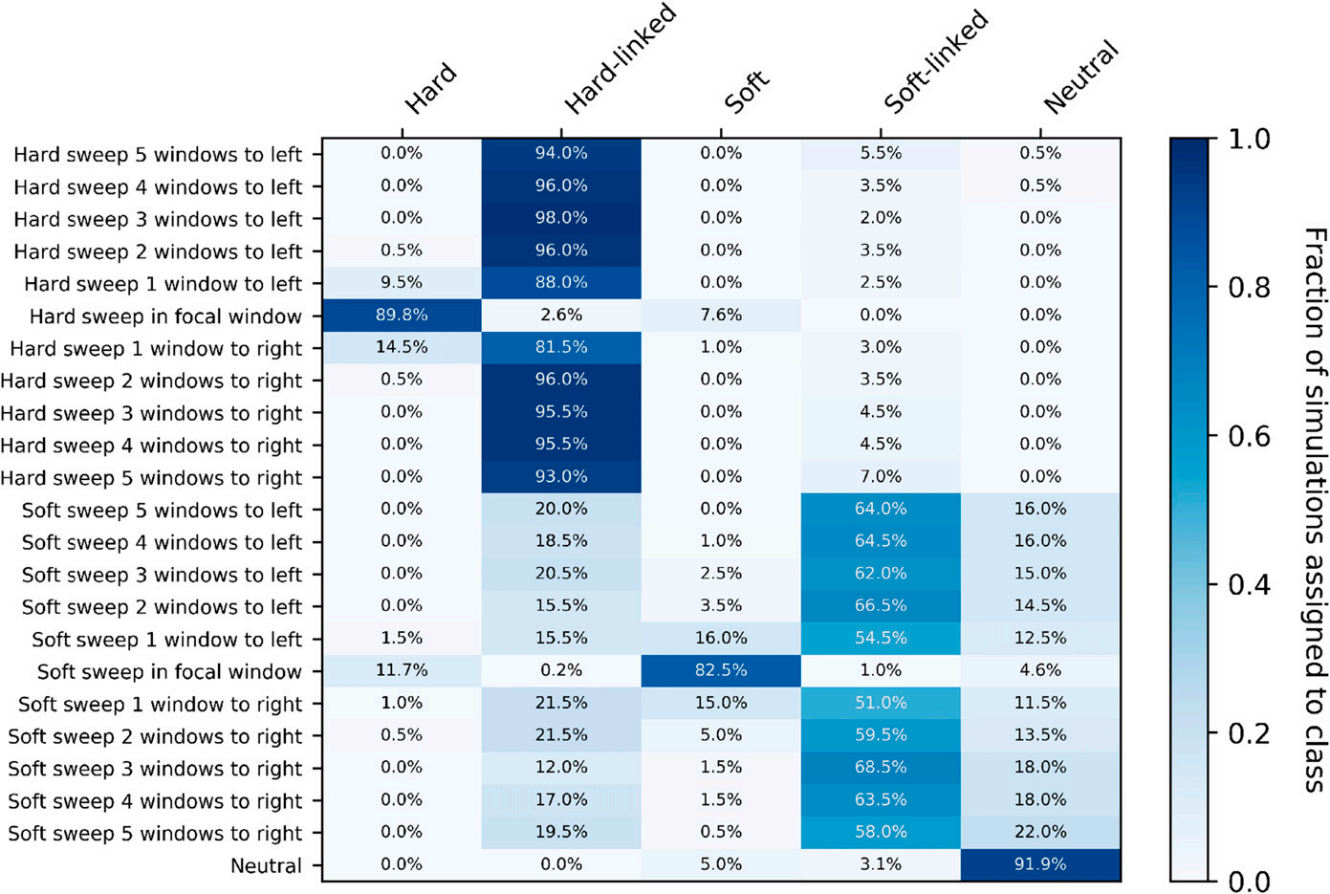
Confusion matrices of subwindows across a recombining chromosome. On the y-axis the location of the classified subwindow relative to the sweep is shown while the x-axis shows the predicted class for each subwindow from diploS/HIC. These results are from simulations using the same parameter set as in Figure 6, using a sample size of n=60 haploid chromosomes.

ROC curves show one aspect of our diploS/HIC classifier, but perhaps more illuminating is the examination of classification in a spatial context (*i.e.*, along a recombining chromosome). To do this we visualize the confusion matrix for 10 subwindows surrounding a central subwindow that has undergone either a hard or soft sweep. Simulated subwindow examples are then classified as belonging to one of five classes (hard sweep, soft sweep, linked to a hard sweep, linked to a soft sweep, or neutral) and the fraction of each such classification is recorded. Figure 7 shows such a confusion matrix for a constant size population with moderately strong selective sweeps, α∼U(250,2500), and for an intermediate sample size of n=60 haploid chromosomes. For this sample size and parameterization diploS/HIC performs quite similarly to our original haploid S/HIC implementation with greater than 86% of sweep windows identified correctly and with a false positive rate of ∼5%. The original S/HIC had a both a slightly higher accuracy on sweeps (∼87.5%) and false positive rate (∼6.7%) on the same test scenario (but with a slightly smaller sample size of n=50; see Schrider and Kern (2016) Figure 4).

**Figure 8:**
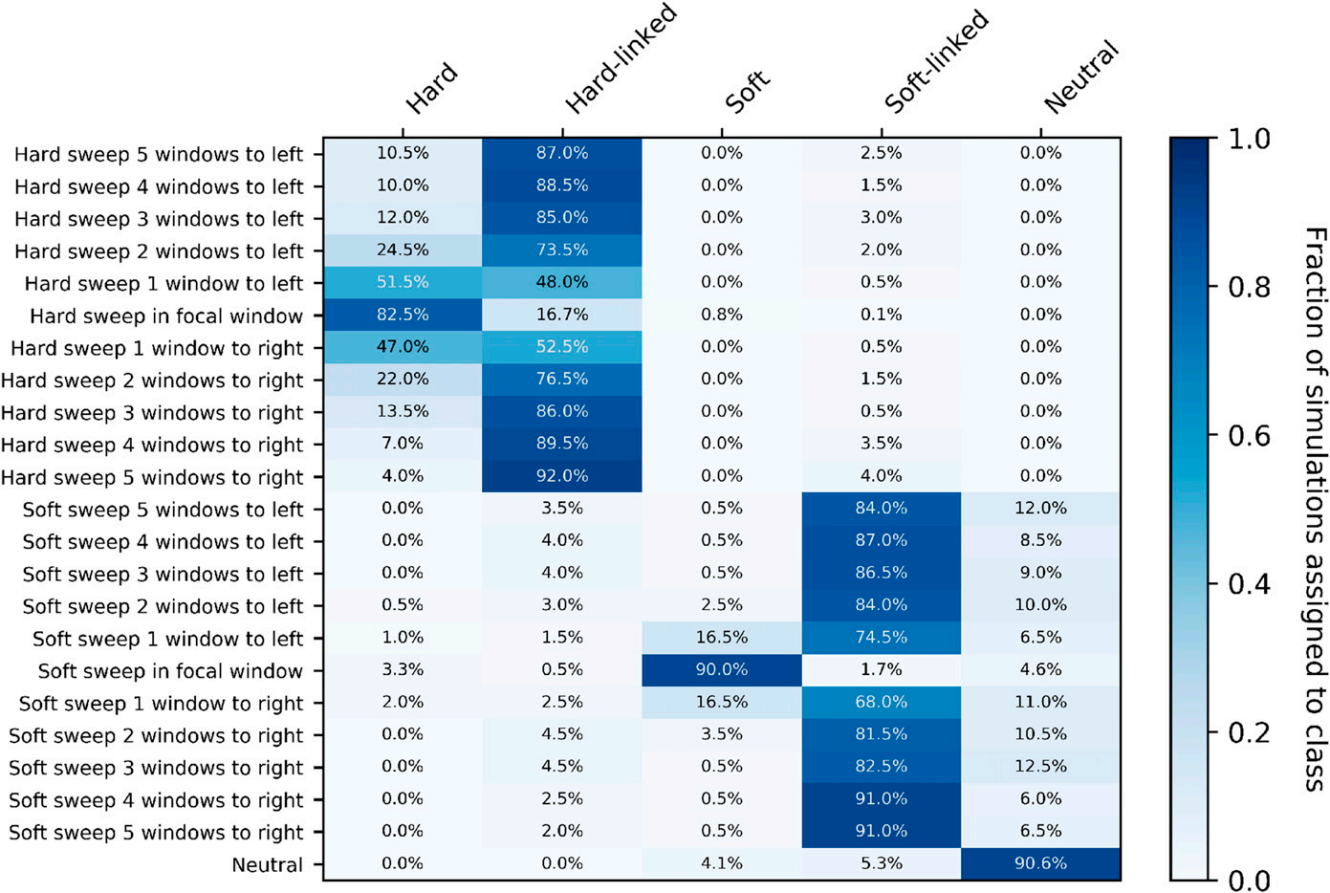
Confusion matrices of subwindows across a recombining chromosome with stronger selection. On the y-axis the location of the classified subwindow relative to the sweep is shown while the x-axis shows the predicted class for each subwindow from diploS/HIC. These results are from simulations using the same parameter set as in Figure 6 with the exception of selection which is an order of magnitude stronger, α∼U(2500,25000), and again using a sample size of n=60 haploid chromosomes.

For stronger sweeps we see qualitatively similar patterns, with an excellent ability to localize both hard and soft selective sweeps (Figure 8). Simulating sweeps with α∼U(2500,25000) but holding constant the rate of recombination means that a greater portion of the chromosome is affected by the sweep. We can see this in our increased misclassification rate of subwindows closest to hard sweeps in Figure 8 relative to Figure 7. Nevertheless our multiclass accuracy (the prediction accuracy across all data classes) on stronger sweeps is still high (∼94%; Figure S2).

On the other hand, when selection is comparatively weak, a relatively smaller portion of the chromosome is perturbed by a selective sweep and thus sweep signatures can be harder to detect, but somewhat easier to localize if detected. In Figure S6 we show confusion matrices associated with an order of magnitude weaker selection, using α∼U(25,250). In this scenario we have reduced sensitivity to sweeps, a slightly higher false positive rate, and struggle to determine the mode of selection. With weak selection diploS/HIC is less likely to identify flanking regions as sweeps, though we struggle with classification of hard-linked and soft-linked regions, misclassifying each type both as the other, or often as neutral in the case of soft-linked sites; such errors are of minor concern if one’s primary goal is the discrimination between sweeps and unselected regions. Supporting this point is the fact that our binary classifier (sweep *vs.* linked+neutral) has a high area under the curve (AUC; ∼0.96) even though our multiclass accuracy is lower (∼64%; see Figure S3); thus even with very weak selection our classifier can find sweeps.

**Figure 9:**
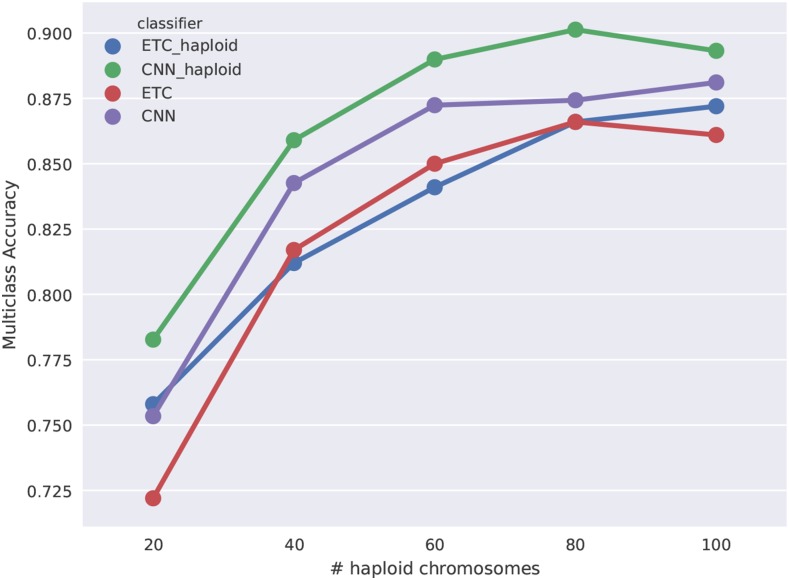
Multiclass classification accuracy for diploS/HIC and related classifiers for *Anopheles* demographic history with moderate selection. Here we compare the multiclass classification accuracy of four related classifiers across a variety of sample sizes: the original S/HIC classifier (“ETC_haploid”), a S/HIC classifier which uses a CNN rather than an ETC (“CNN_haploid”), our new classifier for unphased data trained with an ETC (“ETC”), and finally our new classifier training with a CNN (“CNN”).

**Figure 10:**
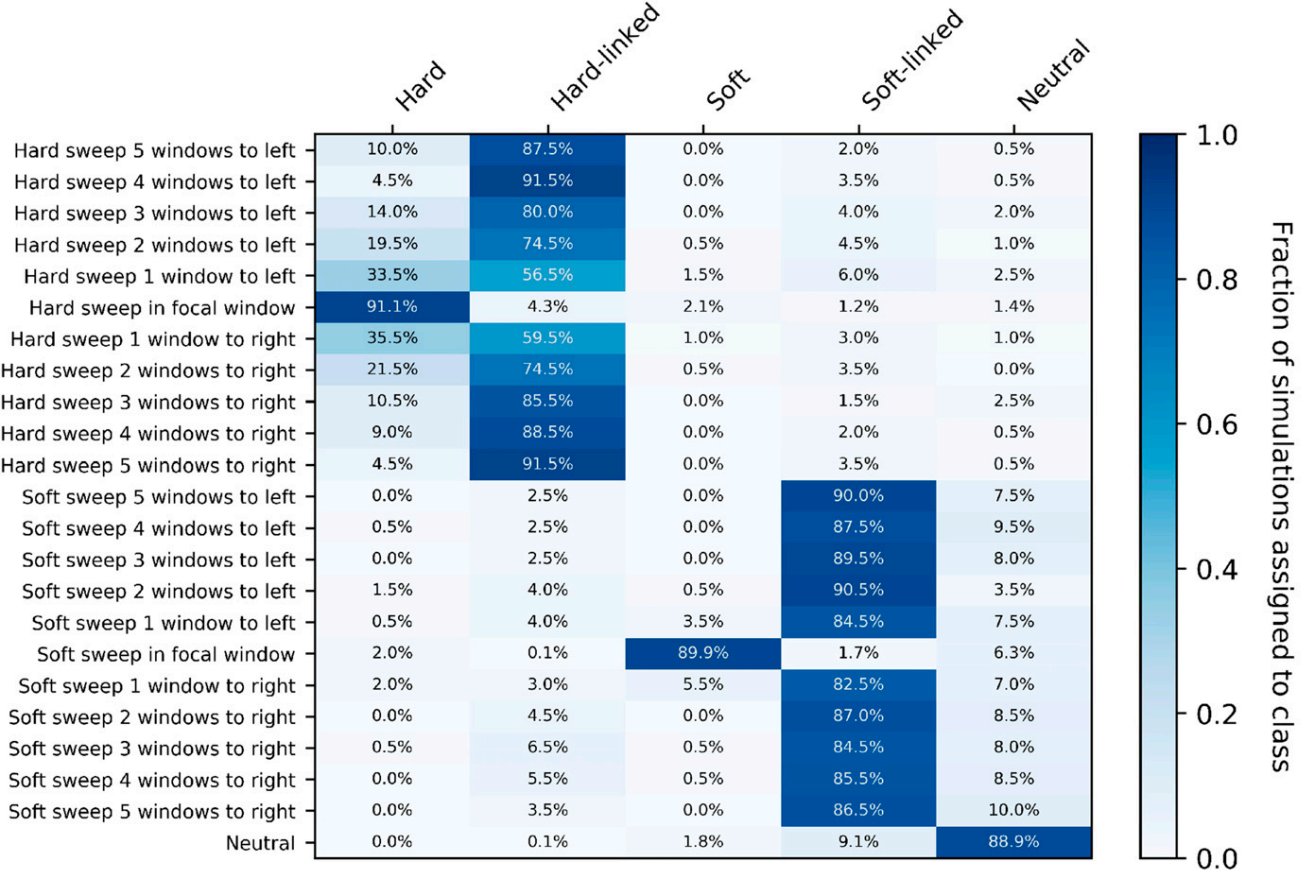
Confusion matrices of diploS/HIC for an *Anopheles gabiae* demographic history. On the y-axis the location of the classified subwindow relative to the sweep is shown while the x-axis shows the predicted class for each subwindow from diploS/HIC. These results are from simulations using the parameters reflecting the population history of a BFS sample of *Anopheles gambiae*, using a sample size of n=60 haploid chromosomes. Here we have simulated sweeps with moderate selection coefficients, α∼U(250,2500).

Each of the above scenarios simulated populations with constant population size, which is a rarity in the natural world. We thus turn attention to the more realistic case in which population size has changed in recent history. In particular we sought to characterize the performance of diploS/HIC for samples from populations with strong growth, such as we recently observed in a Burkino Faso population of *Anopheles gambiae* (Anopheles gambiae 1000 Genomes Consortium (2017)). We inferred using information from the site frequency spectrum that this sample, shorthanded BFS, had experienced greater than 30-fold growth in the past 100,000 years (Anopheles gambiae 1000 Genomes Consortium (2017)). We simulated training sets under the BFS sample’s inferred demographic parameters (see Table S1) for a variety of sample sizes and selection scenarios. Figure 9 shows the multiclass accuracy of diploS/HIC (labeled CNN) and related classifiers when testing against an independent set of simulations. As in the case of constant population size, the general trend is that CNN-based classifiers consistently outperform those based on Extra-Trees. Moreover in this case diploS/HIC even tends to outperform our original implementation of S/HIC which uses phased haplotypes. Supplementary Figures S7 and S8 show the same comparisons for when selection is an order of magnitude stronger and weaker respectively. Under this demographic scenario in our stronger selection case, α∼U(25000,250000), selection is strong relative to recombination and we lose the ability to localize hard sweeps, resulting in a loss of accuracy. On the contrary our weak selection scenario for *Anopheles* yields excellent multiclass accuracies. As before, we prefer to visualize performance using confusion matrices, and in Figure 10 we show representative classifications as a function of distance from each sweep type for simulations of BFS demography and moderately strong sweeps. With n=60 haploid chromosomes we have 87% accuracy and our ability to localize sweeps is quite good, although selection in this case is sufficently strong relative to recombination such that we have a significant misclassification rate of windows neighboring hard sweeps into the hard sweep category. If selection is an order of magnitude weaker for BFS demography then our localization for those windows neighboring sweeps improves considerably (Figure S10). However, if selection is an order of magnitude stronger diploS/HIC and related classifiers all struggle to accurately find hard sweeps, as for this parameterization selection is too strong relative to recombination (Figure S9), therefore larger window sizes would be required.

We previously showed our S/HIC classifier to be quite robust to demographic model misspecification (Schrider *et al.* (2016)), thus we were curious to see if our new implementation should share similar properties. *A priori* we expect diploS/HIC to be robust, as such robustness stems from the summary statistic transform that we apply to our input statistics, rather than some higher level property of the ML algorithm. To characterize accuracy in the face of model misspecification we used the classifiers that we had trained on constant size population simulations (*e.g.*, Figure 5) to make predictions from data drawn from the BFS sample demographic model (*i.e.*, strong population growth). In Figure S11 we show ROC curves for both our original S/HIC and diploS/HIC with and without model misspecification for each of a set of five sample sizes. Both S/HIC and diploS/HIC are indeed robust to model misspecification and while S/HIC is more accurate, owing to its use of haploid information, diploS/HIC is more robust to such misspecification.

Practicing population geneticists often are faced with data can only be phased computationally, rather than through transmission or inbreeding. We were thus interested in characterizing the performance of our classifiers in the face of phasing switch errors. To explore this, we trained haploid and diploid diploS/HIC classifiers, along with S/HIC classifiers on data that contained no switch errors, but then tested those trained classifiers on data which had switch errors inserted at various probabilities (*i.e.*, genotypes at heterozygous sites were flipped at a given rate). In Figure S12 we show the accuracy of three classifiers as a function of switch error rate. Those classifiers are the original S/HIC classifier (labeled “ETC_haploid”), a CNN classifier which uses the original S/HIC feature vector (“CNN_haploid”), and our unphased diploS/HIC classifier (labeled “CNN”). As expected the accuracy of the unphased classifier does not change with increasing switch error rate, however both of the classifiers that make use of phase information do lose accuracy. Thus if there is reason to believe that computational phasing might lead to appreciable error rates we recommend that the user should use the default, unphased version of diploS/HIC.

In general we find that under a more complex history of population growth, such as that from the BFS sample, diploS/HIC performs quite well at finding both hard and soft sweeps and differentiating among them. Moreover we find that diploS/HIC is quite robust to model misspecification during training. This is the case even for reasonably small population sizes, although our smallest sample size tested did lag in accuracy considerably. Thus a fair degree of caution should be taken when analyzing smaller samples (*i.e.*, n≤20) using our method.

### An application to Anopheles gabiae genome sequence data

**Figure 11:**
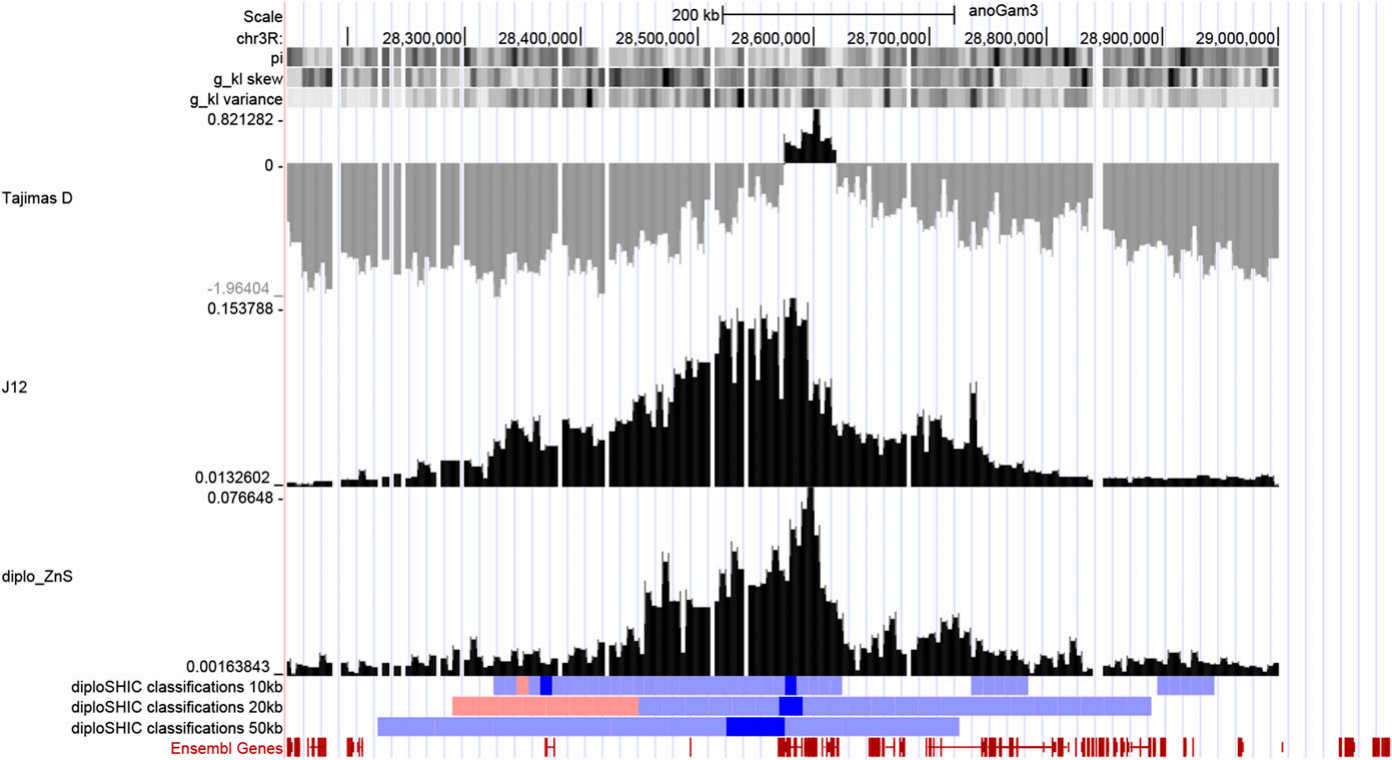
A large soft sweep at *Gste* in *Anopheles gambiae*. Here we show diploS/HIC classifications at three physical scales along with associated summary statistics for a megabase region surrounding the *Gste* cluster on chromosome 3R for the BFS sample. diploS/HIC classifications are colored here such that windows classified as soft sweeps are dark blue, linkedSoft as light blue, and linkedHard as light red. Above the diploS/HIC classifications we show a subset of the summary statistics used as part of the feature vector (see Methods for details).

As an example of how diploS/HIC might be used to elucidate the evolutionary history of an organism we here provide a targeted look at the *Gste* region of the *A. gambiae* genome. Over the last 20 years as part of the Roll Back Malaria initiative, mosquito populations have been subject to large-scale application of insecticides and as a consequence have begun to adapt to this new environment through the evolution of resistance (Hemingway *et al.* (2016)). The *Gste* locus contains a cluster of glutathione S-transferase genes including *Gste2*, which has previously been shown to be involved in detoxification of pyrethroids and DDT (Mitchell *et al.* (2014)). In Figure 11 we show diploS/HIC classifications of windows throughout the region at three physical scales (10kb, 20kb, and 50kb). For computational convenience, we trained a classifier using simulations of genetic distances that correspond to ∼5kb, but predict on feature vectors from a larger region. Though ideally simulation with a matching window size should be used, this is acceptable for our purposes here because our feature vector itself is scale free, and the ratio of the recombination rate to the selection coefficient is what determines the spatial patterns of variation across a region. At each scale a soft sweep is identified at the *Gste* cluster, and one can see from patterns of Tajimas’s *D*, $J_{12},$ and ZnS, that the signal of selection extends for a long distance in each direction away from the sweep. This squares nicely with the haplotype based analysis done by Anopheles gambiae 1000 Genomes Consortium (2017) which showed that numerous haplotypes were under selection at *Gste*. We have included the workflow of this entire empirical application as an example use case of diploS/HIC here: https://github.com/kern-lab/diploSHIC/wiki/A-soup-to-nuts-example.

## Discussion

Creating a complete catalog of selective sweeps in the genome of any organism remains an elusive goal. Population genetics, as a field, requires knowledge of the targets of selection for at least two separate purposes. At a broad scale, we would like to know about genome-wide parameters such as the rate of selective sweeps per generation and their associated distribution of selective effects. At a finer scale, we would like to know the precise location of individual beneficial fixations, so that we can learn about the functional ramifications of adaptive evolution. Thus through the creation of a catalog of selective sweeps we hope to learn both about the process of adaptation as well as the nature of the adaptations themselves.

While methods development for sweep finding tools has a rich history in the literature, in the past few years substantial performance gains have come through leveraging supervised machine learning approaches (reviewed in Schrider and Kern (2018)). In particular, the use of machine learning has led to more powerful methods for detecting sweeps that are robust to complicated demographic histories which had confounded earlier approaches. While this is so, nearly all machine learning methods for finding selection require the use of phased haplotype information for input (the sole exception being SFSselect (Ronen *et al.* (2013)), which is based strictly on the site frequency spectrum). In this report we present a new method that we call diploS/HIC that has exceptional power for finding both hard and soft selective sweeps and differentiating among them without requiring phased data. diploS/HIC builds on the strategy of our earlier method, S/HIC (Schrider and Kern (2016)), in utilizing a large vector of summary statistics that have been transformed to capture spatial information around a focal region to be classified. For diploS/HIC we have introduced a number of new population genetic summary statistics that are compatible with unphased genotypes and which are useful for detecting and discriminating between hard and soft selective sweeps. Note that, like the original S/HIC, diploS/HIC was designed with completed sweeps in mind, but could be extended to handle incomplete sweeps in a straightforward manner by simulating appropriate training data and including additional summary statistics.

Algorithmically diploS/HIC utilizes a deep convolutional neural network (CNN) as the basis of its classification (LeCun *et al.* (2004); Krizhevsky and Hinton (2010); Krizhevsky *et al.* (2012)). Thus, we cast the sweep finding problem as one of image recognition. Our goal in doing so is to more explicitly utilize the spatial covariance structure of summary statistics in a given region, beyond the simple spatial transformation we had been using in Schrider and Kern (2016). While it seems this has succeeded to some degree (*i.e.*, we see that a CNN classifier using the S/HIC feature vector outperforms our original implementation – Figure 5) we suspect that there is still considerable room for improvement. First, the use of recurrent neural network approaches that are specifically formulated for capturing signals in sequential data should provide a principled way of gleaning further spatial signal using summary statistics (Graves *et al.* (2013); Sutskever *et al.* (2014)). Second, we imagine that improvements in using the full information in the spatial arrangement of polymorphism surrounding a sweep could come from abandoning summary statistic representations of loci, and instead using image recognition (*i.e.*, CNN) approaches on images created directly from sequence alignments of a genomic region (Chan *et al.* (2018); L. Flagel, personal communication). A similar approach using images of read pileups was recently introduced for variant calling, yielding solid performance improvements over competing methods (Poplin *et al.* (2017)).

In summary we have shown that diploS/HIC performs quite well when compared to our previous S/HIC under both simple and more complex population size histories. Identifying selective sweeps in populations with non-equilibrium demographic histories remains an important and difficult problem, particularly in cases where the underlying demographic model is unknown or poorly estimated. For instance it is well known that certain demographic models, such as population growth, can mimic selective perturbations (Simonsen *et al.* (1995); Jensen *et al.* (2005)). Here we have shown as with S/HIC before it, diploS/HIC is accurate in the face of non-equilibrium demography, even when misspecified. In conclusion, we believe that diploS/HIC provides yet another powerful tool for population geneticists to use when phased information is unavailable.
